# 局限期小细胞肺癌合并胸水临床特点及预后因素分析

**DOI:** 10.3779/j.issn.1009-3419.2018.01.03

**Published:** 2018-01-20

**Authors:** 昆鹏 许, 优优 王, 静 齐, 路军 赵, 平 王

**Affiliations:** 300060 天津，天津医科大学肿瘤医院放疗科，国家肿瘤临床医学研究中心；天津市“肿瘤防治”重点实验室；天津市恶性肿瘤临床医学研究中心 Department of Radiation Oncology, Tianjin Medical University Cancer Institute and Hospital, National Clinical Research Center for Cancer; Key Laboratory of Cancer Prevention and Therapy, Tianjin; Tianjin's Clinical Research Center for Cancer, Tianjin 300060, China

**Keywords:** 局限期, 小细胞肺癌, 胸腔积液, 预后, Limited-stage, Small cell lung cancer, Pleural effusion, Prognosis

## Abstract

**背景与目的:**

一般将含有肿瘤细胞的胸水称为恶性胸水，预后差，因量少而无法定性胸水预后同样较差。本研究分析合并胸腔积液局限期小细胞肺癌患者的临床特点、预后因素。

**方法:**

回顾性分析了2007年10月-2016年1月期间于我院诊断为局限期小细胞肺癌542例患者，对其中初诊含有胸水患者109例的临床特点及生存时间、预后因素进行分析。主要观察指标为总生存期及无进展生存期。

**结果:**

全组患者中位总生存期、中位无进展生存期分别为29.4个月、18.2个月。治疗前合并胸水的患者较无胸水者生存期明显缩短（中位OS 21.0个月*vs* 31.7个月，中位PFS 14.1个月*vs* 19.1个月；*Log-rank* *P*=0.001, *P*=0.014），*Cox*多因素分析表明胸水是局限期小细胞肺癌的独立预后因素（*P*=0.004）；对于胸水患者，单因素分析提示影响OS的因素有临床分期、淋巴结分期、KPS评分、肺不张、治疗后胸水状态；而*Cox*多因素分析提示，治疗后胸水状态是合并胸水的局限期小细胞肺癌患者的独立预后因素（*P*=0.016）；患者初治无胸水（*n*=433）与经治后胸水消失（*n*=67）、胸水未消失（*n*=32）三组患者中位OS分别为31.7个月、23.2个月和16.8个月，中位PFS为19.1个月、17.9个月和11.4个月，三组生存期之间比较有明显差异（*Log-rank*, *P* < 0.001, *P* < 0.002），其中后两组差距明显（*Log-rank*, *P*=0.046, *P*=0.013），而前两组比较无明显差异（*Log-rank*, *P*=0.088, *P*=0.656）；对于胸水的患者，行放化疗治疗与单独化疗相比并无明显差距（*Log-rank*, *P*=0.243, *P*=0.390）。

**结论:**

合并胸水局限期小细胞肺癌生存期明显缩短，经治疗后胸水消失是生存的独立良好预后因素，如何治疗值得进一步探索。

## Abstract (en)

**Background and objective:**

Malignant pleural effusion (PE) was generally defined as pleural effusion containing tumors with poor prognosis. Some kinds of undefined pleural effusions due to too small amount of effusion had poor prognosis too. This study aimed to analyze the clinical characteristics and prognostic factors of patients who suffered from limited-stage small cell lung cancer (LS-SCLC) complicated with pleural effusion.

**Methods:**

A retrospective analysis included 542 patients who were diagnosed with LS-SCLC and had treatment in our hospital from October 2007 to January 2016. We had observed 109 patients who were diagnosed with pleural effusion at their first visit to the doctor. We analyzed the clinical characters, survival time and the prognostic factors of the 109 patients. Our main observation targets were overall survival (OS) and progression free survival (PFS).

**Results:**

The median OS and PFS of whole group were 29.4 and 18.2 months. Before treatment, survival time of patients with PE were significantly shorter than patients without PE (median OS: 21.0 *vs* 31.7 months; median PFS: 14.1 *vs* 9.1 months; *Log-rank*, *P*=0.001, *P*=0.014). Multi-factor analysis of multivariate *Cox* shows PE was the independent prognostic factor of LS-SCLC (*P*=0.04). Single factor analysis showed factors affecting PE patient's survival time included clinical stages, lymph node (LN) stages, KPS scores, pulmonary atelectasis and the state of pleural after treatment. *Cox* multi-factor analysis reminded that the state of pleural effusion after treatment was the independent prognostic factor of LS-SCLC complicated with pleural effusion (*P*=0.016). There were three groups was apportioned patients without pleural effusion before treatment (group 1; *n*=433), patients whose pleural effusion disappeared after treatment (group 2; *n*=67) and patients whose pleural effusion didn't disappear after treatment (group 3; *n*=32).The median OS were 31.7, 23.2, 16.8 months in the group 1, 2, 3 and the median PFS were 19.1, 17.9, 11.4 months. Obvious difference was noted by the comparison of survival time of these three groups (*Log-rank* *P* < 0.001, *P* < 0.002). The difference between group 2 and group 3 was significant (*Log-rank* *P*=0.046, *P*=0.013) while no obvious difference was noted during comparison of group 1 and group 2. For patients who have LS-SCLC complicated with PE, there is no remarkable difference between chemoradiotherapy and chemotherapy alone.

**Conclusion:**

The survival time of patients who suffered from limited-stage small cell lung cancer complicated with pleural effusion was obviously shortened. The disappearing of pleural effusion after treatment was the independent favorable prognostic factor of survival. How to treat needed further investigation.

小细胞肺癌（small cell lung cancer, SCLC）约占原发肺癌患者的13%^[1]^，相对于非小细胞肺癌，具有恶性程度高，进展快，易发广泛转移的生物学特点。临床上常采用美国退伍军人医院两期法和国际抗癌联盟（Union for International Cancer Control, UICC）肺癌TNM方法进行分期。通常将含有肿瘤细胞的胸水称为恶性胸水，TNM分期归为M1a、Ⅳ期^[2]^。1989年国际肺癌协会（International Association for the Study of Lung Cancer, IASLC）共识，将胸水不管恶性还是良性，均归为局限期^[3]^。而2007年第七版TNM分期究发现含有恶性胸水的SCLC患者生存期介于局限期和广泛期无胸水的患者之间^[4]^。对于胸水过少的患者无法定性诊断时，胸水一般就无法进行适当地分期归类。研究人员发现胸水（pleural effusion, PE）的存在影响患者的生存时间，即使是少量，也是肺癌预后的不良因素^[5-8]^。我们的研究结果同样表明对于局限期SCLC，含有胸水的患者生存期明显缩短，是预后不良的独立因素，不过经治疗后胸水消失的患者生存时间明显获得延长，本研究回顾性分析本院542例局限期小细胞肺癌（limited-stage small cell lung cancer, LS-SCLC）中治疗前合并胸水患者109例临床及预后特点，并对胸水患者进行了相关预后因素分析。

## 1 材料和方法

### 1.1 临床资料

回顾分析了2007年10月-2016年1月期间在天津医科大学肿瘤医院初诊为局限期SCLC（美国退伍军人医院分期标准）患者，共542例，其中经影像学证实，初治时合并胸水患者为109例。所有患者均经病理学或细胞学证实为SCLC且按照（UICC 2009年）肺癌TNM分期重新进行临床分期，见表 1。

**1:** 患者一般临床资料 Patients characteristics

	Total number of patients (*n*=542)	PE (*n*=109)	No PE (*n*=433)	*P*
Age (yr)	58 (25-83)	58 (39-80)	60 (25-83)	0.132
≥65	169	27	142	
< 65	373	82	291	
Gender				0.077
Male	385	85	300	
Female	157	24	133	
Smoking				< 0.999
No	163	33	133	
Yes	379	76	303	
Smoking indexes				0.515
≥400	317	67	250	
< 400	225	42	183	
KPS at diagnosis				0.116
≥80	598	96	402	
< 80	44	13	31	
Weight loss				0.194
No	452	86	366	
Yes	90	23	67	
N				0.006
N0	87	6	81	
N1	57	10	47	
N2	310	71	239	
N3	88	22	66	
Staging				0.073
T1N0	40	4	36	
T2-3N0, T1-2N1	59	8	51	
T1-2N2, T3N1-2, T4N0-1	308	62	246	
T4N2, T1-4N3	135	35	100	
PCI				0.645
Yes	171	32	139	
No	371	77	294	
Treatment				< 0.001
Concurrent radiotherapy	187	45	142	
Sequential radiotherapy	221	36	185	
Surgery	89	5	84	
Chemotherapy	45	23	22	
PE: pleural effusion; PCI: prophylactic cranial irradiation.				

### 1.2 治疗情况和分组

109例含有胸水患者均接受相关治疗，其中83例接受放疗，同步放疗45例，序贯放疗35例，术后放疗3例，单独化疗23例，手术5例；其中107例患者接受不同周期诱导化疗，其中42例接受EC方案化疗，58例接受EP方案，余4例接受其他方案化疗；32例患者于放化疗后接受脑预防照射（prophylactic cranial irradiation, PCI）；经治疗后，67例患者胸水消失，32例仍存在，有5例患者无法评估，5例患者手术；只有4例患者进行胸水病因学检测，均未检测到肿瘤细胞，其他病人因胸水量过少无法进行穿刺，所有患者均未进行相应的局部胸水治疗；其中5例患者为双侧少量胸水，余均与患者肿瘤同侧。

### 1.3 随访和观察终点

随访截止2017年5月1日，其中259例（47.9%）患者死亡。总生存期：从病理确诊日期开始至患者死亡或末次随访日期；无进展生存期（progression-free survival, PFS）：从病理确诊开始至肿瘤出现影像学证实的进展复发或末次随访日期。

### 1.4 统计学方法

采用SPSS 23.0软件进行统计学分析，组间资料比较采用卡方检验，运用*Kaplan-Meier*法进行单因素预后分析，*Cox*模型进行多因素预后分析，*P* < 0.05为差异具有统计学意义。

## 2 结果

### 2.1 患者生存时间

全部患者中位OS为29.4个月，1年、3年的生存率分别为87.2%、41.0%，中位PFS为18.2个月，1年、3年的PFS生存率为66.0、37.0%，合并胸水患者中位OS、中位PFS分别为21.0个月、14.1个月，而初治时无胸水的患者为31.7个月、19.1个月，有、无胸水的OS、PFS有明显差距（*P*=0.001, *P*=0.014），见表 2。

**2:** 患者生存生存时间数据 Survival time of patients

	OS				PFS		
	Median OS （mo 95%CI）	1-yr survival rate（%）	3-yr survival rate（%）		Median PFS (mo 95%CI)	1-yr survival rate（%）	3-yr survival rate（%）
All patients	29.4 (25.4-33.4)	87.2	41.0%		18.2 (15.6-20.7)	66.0	37.0
With PE	21.0 (16.5-25.6)	81.6	24.5		14.1 (11.8-16.1)	58.7	25.1
Group 1	23.2 (18.6-27.8)	86.1	29.6		17.9 (13.2-22.7)	65.3	30.0
Group 2	16.8 (14.9-18.7)	74.4	8.1		11.4 (5.6-17.3)	46.8	10.0
Without PE	31.7 (27.9-35.1)	88.1	44.3		19.1 (15.6-22.7)	67.2	39.2
*By *Log-rank test*. CI: confidence interval; Mo: months; OS: overall survival; PFS: progression-free survival; Group 1 pleural effusions disappearing after treatment; Group 2 without pleural effusions disappearing after treatment.										

### 2.2 SCLC预后因素分析

将影响局限期SCLC患者的单因素分析结果中*P* < 0.20（年龄、吸烟、吸烟量、PCI、分期、KPS评分、治疗方案）临床因素纳入*Cox*多因素分析，结果提示胸水是局限SCLC的独立预后因素（*P*=0.005），见表 3。

**3:** 局限期小细胞肺癌患者*Cox*模型多因素分析 Results of analysis of survival factors of overall small cell lung cancer patients

Survival factors	Univariate analysis		Multivariate analysis		
	*P*		*P*	HR	95% CI
Age (≥65 *vs* 65)	0.049		0.339	1.13	0.87-1.46
Smoking	0.001		0.259	1.28	0.83-1.98
Smoking index (≥400 *vs* < 400)	< 0.001		0.467	1.16	0.77-1.71
KPS (≥80 *vs* < 80)	0.056		0.297	1.23	0.83-1.83
Staging	< 0.001		< 0.001	1.96	1.35-2.87
PE (Yes *vs* No)	< 0.001		0.005	1.52	1.15-2.06
PCI (Yes *vs* No)	< 0.001		< 0.001	0.55	0.42-0.72
Treatment	< 0.001		0.354	0.93	0.81-1.08
*By *Log-rank* test. KPS: Karnofsky performance status.					

### 2.3 影响胸水患者OS单因素预后分析

纳入分析危险因素依次有：年龄、性别、吸烟、吸烟指数、KPS（Karnofsky performance status）评分、肿瘤位置、肺不张、分期、淋巴结分期、PCI、治疗后胸水状态、治疗方案以及实验室指标，发现KPS评分、有无合并肺不张、TNM分期、淋巴结分期、治疗后胸水状态有明显差异（*P* < 0.05）。同时将*P* < 0.20的变量纳入多变量回归模型中进一步分析，发现治疗后胸水状态是小细胞肺癌合并胸水患者的独立预后因素（*P*=0.016），见表 4-表 6。

**4:** 胸水患者患者资料及单因素分析 Demographic charateristics and survival time of patients

	Median survival time (mo)	95%CI	*P*
Age (yr)			0.216
≥65	21.0	14.3-29.9	
< 65	22.1	15.5-26.5	
Gender			0.564
Male	21.3	18.4-24.3	
Female	20.8	12.9-28.7	
Smoking			0.284
No	24.9	21.9-27.9	
Yes	20.6	16.7-24.5	
Smoking indexes			0.121
≥400	24.9	21.7-28.1	
< 400	20.2	16.4-24.0	
KPS at diagnosis			0.035
≥80	21.9	18.9-25.9	
< 80	13.3	9.4-17.1	
Weight loss			0.470
No	21.3	19.6-23.1	
Yes	17.8	15.0-20.6	
Location of tumor			0.091
Right	20.8	17.1-24.6	
Left	24.9	13.6-36.2	
Pulmonary atelectasis			0.029
No	26.7	19.3-34.2	
Yes	20.2	14.5-25.9	
Staging			0.026
T1-2N1-2, T3N0	35.4	14.5-56.3	
T3N1-3, T4N0-3	20.6	16.5-24.7	
N			0.025
0, 1	35.4	27.9-42.9	
2, 3	20.2	16.5-23.9	
PCI			0.105
No	23.2	16.0-30.4	
Yes	20.6	15.8-255	
Treatment			0.237
Concurrent radiotherapy	21.3	19.1-22.9	
Sequential radiotherapy	21.0	15.3-26.7	
Chemotherapy	17.8	3.9-31.7	
PE disappearing after treatment			0.046
No	16.8	14.9-18.7	
Yes	24.9	19.3-30.5	
*By *Log-rank* test.			

**5:** 实验室相关预后因素 The effect of laboratory data on survival time

	Median survival time (mo)	95%CI	*P*
Blood LDH (U/L)			0.388
≥250	17.4	12.9-23.9	
< 250	21.3	18.5-24.1	
Blood protein (g/L)			0.777
≥110	21.3	16.4-26.3	
< 110	20.2	0.44-40	
Blood albumin (g/L)			0.935
≥35	21.0	16.2-25.8	
< 35	21.0	5.4-36.7	
NSE (*μ*g/L)			0.076
≥50	23.2	18.7-27.8	
< 50	15.4	12.0-18.7	
CYFRA21-1 (*μ*g/L)			0.476
≥3.3	17.8	9.7-25.9	
< 3.3	22.1	19.1-25.1	
*By *Log-rank* test.			

**6:** 多因素分析结果 Results of *Cox* regression for multivariate analysis of survival factors

	HR	95%CI	*P*
Smoking indexes (≥400 *vs* < 400)	1.61	0.86-3.00	0.137
KPS (≥80 *vs* < 80)	1.62	0.80-3.25	0.903
Location of tumor (Right *vs* Left)	0.55	0.29-1.05	0.071
NSE (≥50 *vs* < 50)	1.19	0.59-2.40	0.623
Pulmonary atelectasis (Yes *vs* No)	1.63	0.88-3.04	0.124
PCI (Yes *vs* No)	0.83	0.44-1.52	0.561
Staging	3.38	0.39-27.38	0.503
N (0, 1 *vs* 2, 3)	1.11	0.25-4.87	0.895
PE disappearing after treatment (YES *vs* NO)	0.43	0.22-0.86	0.016
*By *Cox* test.			

### 2.4 治疗后情况

初治无胸水与经治疗后胸水消失、胸水未消失三组患者中位OS分别为31.7个月、23.2个月、16.8个月，中位PFS为19.1个月、17.9个月、11.4个月，三组生存期之间比较有明显差异性（*Log-rank* *P* < 0.001, *P* < 0.002）；其中治疗后胸水消失、胸水仍存在两者有明显差距（*P*=0.046, *P*=0.013），对于无胸水与胸水消失组的患者，虽然OS、PFS缩短，但无明显差距（*Log-rank* *P*=0.088, *P*=0.656），胸水未消失的患者与初治无胸水的之间*Log-rank* *P*均 < 0.001，见表 2、图 1。

**1:**
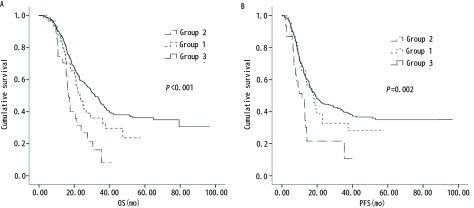
治疗后胸水未消失、消失及初治无胸水三组患者生存曲线图.A：OS；B：PFS。 *Kaplan-Meier* curves of survival time for three groups. Group 1 pleural effusions disappearing after treatment ; Group 2 without pleural effusions disappearing after treatment; Group 3 no pleural effusions before treatment. A: OS；B: PFS.

### 2.5 治疗效果

而对于胸水的患者，放化疗（*n*=81）与单纯化疗（*n*=23）患者的OS、PFS相比并无明显差异（*P*=0.243, *P*=0.390），中位OS分别为21.0个月、17.8个月，中位PFS为13.8个月、17.2个月，进一步对放化疗的患者进行分析，同步放化疗（*n*=45）与序贯放化疗（*n*=36）相比*P*均 > 0.05（*P*=0.942, *P*=0.916），中位OS 21.0个月、21.0个月，中位PFS为13.1个月、14.1个月。

## 3 讨论

本研究结果显示，初诊为为LS-SCLC中合并胸水的患者约20.1%（109/542），高于07年报道的13.0%（145/1, 113），以及2008年日本学者Ryu研究的16.6%（62/373），但低于15年韩国研究学者Niho的27.0%（37/137）^[5, 6]^。初诊时合并胸水的LS-SCLC患者比例约在13.0%-27.0%范围。本研究显示含有胸水患者的中位OS、PFS为21.0、14.1个月，初治时无胸水的患者31.7个月、19.1个月，两组中位OS均高于日本Ryu报道研究（11.8个月、20.9个月），不过该两项研究一致的是，有、无胸水的患者生存期均有显著差异。对于胸水临床因量的缘故无法行穿刺患者，有人把CT上胸水厚度 < 10 mm称为少量胸水，同时也有人把 < 20 mm归于少量，相较无胸水的肺癌患者或恶性胸水患者生存期都有明显差距，甚至有人将一些无法定性的患者也算为少量胸水之中，但结果均一致的说明胸水存在就会影响肺癌患者的生存期，这种所谓”少量胸水”被认为可能是恶性胸水的早期阶段^[5, 7-10]^。

本文对影响SCLC预后的因素进行了多因素分析，提示胸水、分期等是LS-SCLC的独立预后因素。NCCN指南推荐胸水细胞学阳性的患者为恶性胸水，把细胞学阴性归为良性，但胸水量过少无法行细胞学检查，无法明确病因时不作为分期依据。本文研究109例患者只有4例做了病因学检测，均为阴性，其他患者因胸水量过少临床上无法进行胸水穿刺，结果提示合并胸水的患者预后缩短，同其他研究结果一致，均提示无论恶性胸水还是少量胸水均是肺癌的不良预后因素，原因可能是胸水的存在进一步增加了肿瘤的负荷^[5, 10-11]^。

对于LS-SCLC合并胸水单因素分析提示患者临床分期、KPS评分、淋巴结分期、肺不张、胸水治疗后状况*P*值有意义，把这些因素纳入多因素分析，分析提示胸水治疗后消失是患者预后独立因素，表明我们在治疗初要以把这作为治疗目标之一。不难理解，临床分期和淋巴结分期都是基于TNM基础上，所以单因素提示影响预后^[12]^，胸水患者主要分布在较晚期，或者是纵膈淋巴结转移或者N2以上，胸水产生与肿瘤负荷有一定程度的联系。KPS评分表示人体功能状态评分，一般评分越高代表患者身体状况越好，在恶性胸水研究中表明也是人体的功能状况或者体力状况，影响患者的预后^[13]^。肺不张作为肺癌T分期非肿瘤大小的指导分期因素，在一定程度上影响患者预后^[14, 15]^。本研究结果提示合并肺不张的胸水患者预后差，具体原因有待进一步研究。治疗后患者胸水消失的患者生存期明显好于为消失的患者，虽较无胸水患者有所缩短，但无明显差距（*P*=0.088）。对于LS-SCLC合并胸水的治疗暂无大量的循证医学证据，这部分患者也尚无明确治疗方案。Ryu研究表明有对于诱导化疗有效，即治疗后胸水消失的患者，放疗可以延长生存时间，但差异并不明显（*P*=0.196）^[6, 16]^。我们研究结果显示，对于胸水的患者，放化疗与单纯化疗组并无明差异（*P*=0.261），而且同步与序贯相比无明显差异（*P*=0.942），由于单纯化疗病例数据过少，同时未考虑量的因素，仍需更多病例进一步证实。

最后，本文尚存在一些不足之处，未把胸水量纳入分析，所以仍需进一步研究，初诊合并胸水是LS-SCLC患者的不良预后因素，治疗后胸水消失患者生存期明显转归，如何进一步提高这些患者的生存期尚待进一步研究。
